# Supplementary material for: Splice-Junction-Based Mapping of Alternative Isoforms in the Human Proteome
Source: Cell Rep. Author manuscript; Available in PMC 2020 Jan 15. (PMC6961840; doi:10.1016/j.celrep.2019.11.026)
Supplement: 2 [file NIHMS1546469-supplement-2.zip › DF1/PXD009737/TestisTrypsin/TestisTrypsin_30_ABHD12_GCALEHERCAAAGSSSSGSAAAALDADCR.pdf]

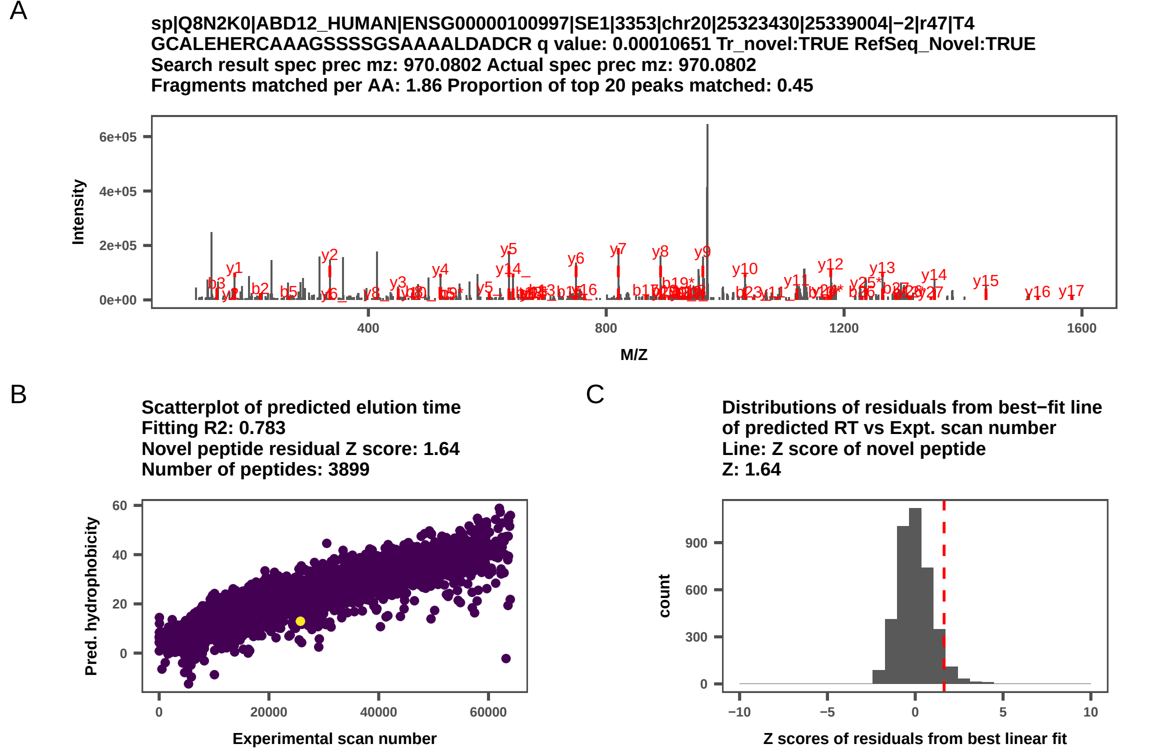
A
sp|Q8N2K0|ABD12_HUMAN|ENSG00000100997|SE1|3353|chr20|25323430|25339004|−2|r47|T4
GCALEHERCAAAGSSSSGSAAAALDADCR q value: 0.00010651 Tr_novel:TRUE RefSeq_Novel:TRUE
Search result spec prec mz: 970.0802 Actual spec prec mz: 970.0802
Fragments matched per AA: 1.86 Proportion of top 20 peaks matched: 0.45
Intensity
6e+05
4e+05
2e+05
0e+00
400
800
1200
1600
M/Z
y1
y2
y4
y5
y6
y7
y8
y9
y10
y11
y12
y13
y14
y15
y16
y17
B
Scatterplot of predicted elution time
Fitting R2: 0.783
Novel peptide residual Z score: 1.64
Number of peptides: 3899
Pred. hydrophobicity
60
40
20
0
0
20000
40000
60000
Experimental scan number
C
Distributions of residuals from best−fit line
of predicted RT vs Expt. scan number
Line: Z score of novel peptide
Z: 1.64
count
900
600
300
0
−10
−5
0
5
10
Z scores of residuals from best linear fit
